# A Review of the Role of the Intestinal Microbiota in Age-Related Macular Degeneration

**DOI:** 10.3390/jcm10102072

**Published:** 2021-05-12

**Authors:** Phoebe Lin, Scott M. McClintic, Urooba Nadeem, Dimitra Skondra

**Affiliations:** 1 Casey Eye Institute, Oregon Health & Science University, Portland, OR 97239, USA; 2 Retina Consultants, LLC, Salem, OR 97302, USA; smcclint@gmail.com; 3 Department of Pathology, University of Chicago, Chicago, IL 60637, USA; Urooba.Nadeem@uchospitals.edu; 4 Department of Ophthalmology, University of Chicago, Chicago, IL 60637, USA; dimitraskondra@gmail.com

**Keywords:** intestinal microbiota, microbiome, age-related macular degeneration, AREDS, complement, diet

## Abstract

Blindness from age-related macular degeneration (AMD) is an escalating problem, yet AMD pathogenesis is incompletely understood and treatments are limited. The intestinal microbiota is highly influential in ocular and extraocular diseases with inflammatory components, such as AMD. This article reviews data supporting the role of the intestinal microbiota in AMD pathogenesis. Multiple groups have found an intestinal dysbiosis in advanced AMD. There is growing evidence that environmental factors associated with AMD progression potentially work through the intestinal microbiota. A high-fat diet in apo-E-/- mice exacerbated wet and dry AMD features, presumably through changes in the intestinal microbiome, though other independent mechanisms related to lipid metabolism are also likely at play. AREDS supplementation reversed some adverse intestinal microbial changes in AMD patients. Part of the mechanism of intestinal microbial effects on retinal disease progression is via microbiota-induced microglial activation. The microbiota are at the intersection of genetics and AMD. Higher genetic risk was associated with lower intestinal bacterial diversity in AMD. Microbiota-induced metabolite production and gene expression occur in pathways important in AMD pathogenesis. These studies suggest a crucial link between the intestinal microbiota and AMD pathogenesis, thus providing a novel potential therapeutic target. Thus, the need for large longitudinal studies in patients and germ-free or gnotobiotic animal models has never been more pressing.

## 1. Introduction 

Age-related macular degeneration (AMD) is a leading cause of irreversible blindness in the developed world, affecting 11 million people in the U.S. and 170 million people world-wide [1,2]. Due to the increasing aging population, the number of AMD-affected individuals is expected to double by the year 2050 [2]. Despite the growing disease burden, our understanding of the pathogenesis of AMD remains incomplete, and treatment options, particularly for non-neovascular disease, remain limited. Advanced AMD is characterized by loss of central vision due to either [1] gradual atrophy of the retina and retinal pigment epithelium (RPE), known as geographic atrophy (GA), or the [2] development of choroidal neovascular membranes (CNVM), which is the defining feature of exudative or neovascular AMD (nvAMD). There are limited therapeutics effective in preventing progression of AMD due to a limited understanding of its pathogenesis. An intersection of genetic and environmental factors likely contribute to AMD pathogenesis. These factors include disruptions in lipid, carotenoid, and inflammatory pathways induced by diet, smoking, and certain gene variants [3,4]. AMD has also been associated with systemic co-morbidities such as obesity, atherosclerosis, and diabetes mellitus [3,5,6]. It has been shown that spouses of subjects who develop AMD were more likely to develop the disease themselves, suggesting that shared lifestyle habits, and potentially, shared microbiota, may increase risk [7].

### 1.1. Inflammatory Mechanisms of AMD

Inflammatory mechanisms of AMD have been described through a series of genetic, immunologic, and other mechanistic studies. These inflammatory mechanisms include aberrations in the complement cascade, inflammasome activation, inflammatory cytokine upregulation, and microglial activation. The complement system is part of the innate immune system that normally aids in the clearance of microbes and injured cells from the body, but when dysregulated can result in immune-mediated diseases. Genetic studies have shown that variants in the complement cascade genes were highly associated with AMD [8], while other studies have shown that several complement activation products have been found in the drusen of AMD eyes [8,9,10] and are increased in AMD patients’ sera [10]. Inflammasomes are intracellular protein complexes that respond to pattern-recognition receptors released during microbial infection or as a result of cellular damage. The nucleotide-binding oligomerization domain (NOD)-like receptor, pyrin domain-containing protein 3, or the NLRP3 inflammasome, is upregulated in the RPE of AMD patients with GA, and is likely activated by components found in the drusen [11,12]. Inflammatory cytokines such as interleukin-6, -17, and -18 have been found to be elevated in the eyes of patients with AMD, but their roles in pathogenesis are unclear [11,13,14,15]. Microglia are specialized myeloid-derived cells usually found within the inner retina. Activated microglia and macrophages have been found in the subretinal space of eyes with AMD [16,17,18], but again, their role in pathogenesis is poorly understood.

### 1.2. The Commensal Microbiota in Regulation of Inflammation and Lipid Metabolism

The commensal microbiota, or microorganisms that normally inhabit the human body, potentially provide a missing link between the genetic and environmental factors that contribute to AMD pathogenesis. The vast majority of the human commensal microbiota is composed of bacteria located in the gastrointestinal tract, which are now known to be integrally involved in host biological processes such as control of the immune system, breakdown of lipids found in the diet, and the formation and metabolism of bile acids. Metabolites and other components produced by or expressed on the commensal bacteria have wide-spanning functions, including modifying host leukocyte trafficking within the adaptive immune compartment and activating crucial innate pathways within the gut, such as complement and the NLRP3 inflammasome [11]. Because of these integrated functions with the host, an intestinal dysbiosis, or alterations in the balance of commensals favoring inflammation or disease, has been demonstrated in several diseases, including type 1 diabetes, atherosclerosis, inflammatory bowel disease (IBD), rheumatoid arthritis (RA), multiple sclerosis (MS), and, recently, AMD [19,20,21,22,23,24]. 

The pathogenesis of AMD may be similar to that of other age- and lifestyle-associated inflammatory diseases. A mechanism has been proposed to link atherosclerosis to the activity of bacteria in the human gut. Phosphatidylcholine obtained through the diet is metabolized by gut bacteria to form trimethylamine oxide (TMAO), and serum levels of TMAO have been linked to atherosclerosis and cardiovascular risk [25]. There is also evidence for an association between intestinal dysbiosis and other ocular diseases. Uveitis is characterized by intraocular inflammation that may be related to systemic inflammatory disease or, when seen in isolation, is presumed to be idiopathic. Alterations to the microbiota have been identified in humans with non-infectious uveitis [26], uveitis-associated systemic conditions (e.g., IBD, RA, MS, ankylosing spondylitis) [21,22,23], and in animal models with experimentally induced uveitis [27,28,29]. Evidence for multiple pathogenic mechanisms has been reported, including microbe-mediated imbalances in effector versus regulatory T cell populations [28,29], decreased bacterial production of anti-inflammatory metabolites such as short chain fatty acids [28], improper sensitization of T cells to self-antigens in response to cross-reactivity with microbial antigens [30], and increased intestinal permeability, which may cause immune over-activation by allowing microbial antigens to enter the systemic circulation [27]. Similarly, glaucoma development may be mediated by T cell sensitization from commensal microbes, highlighted by decreased glaucomamatous neurodegneration in the absence of the microbiota [31]. Evidence has emerged that there may be an association between the gut microbiome and diabetic retinopathy, as well as retinopathy of prematurity, and VEGF-driven angiogenic retinal disorders such as neovascular AMD. Intermittent fasting prevented diabetic retinopathy by restructuring the gut-microbiome in db/db mice [32]. Skondra and colleagues described an increased abundance of *Enterobacteriaceae* species in the early gut microbiome of high-risk premature infants, who later developed severe retinopathy of prematurity needing treatment compared to high-risk infants that did not develop any retinopathy of prematurity [33]. While these mechanisms have not been investigated in AMD, they are avenues of potential analogy and discovery in AMD.

The authors hypothesize that the composition and function of gut bacteria may also modulate immune response in AMD. Because of these functions and what we currently understand about AMD development, intestinal microbiota are probable candidates for the interaction between environmental and inherited influences associated with AMD. This review highlights pre-clinical and clinical data that support the potential impact of alterations in the intestinal microbiota on AMD development or progression.

## 2. Intestinal Dysbiosis in Advanced AMD

Studies showing that an intestinal dysbiosis occurs in advanced AMD have revealed specific bacteria that are differentially abundant in healthy older adults compared to those with advanced AMD. In a study of 12 new exudative AMD patients compared with 11 control subjects, Zinkernagel and colleagues demonstrated differences in the intestinal microbiota [34]. The study reported enriched abundances of the bacterial genera *Anaerotruncus*, *Oscillibacter*, *Ruminococcus torques*, and *Eubacterium ventrosium*. In a different clinical case-control study (85 advanced AMD vs. 49 healthy control subjects), Lin and colleagues discovered an intestinal dysbiosis resulting in enhanced abundance of *Prevotella*, *Holdemanella*, *Desulfovibrio,* and other bacteria, but reduced abundance in *Oscillospira*, *Blautia*, and *Dorea*, in AMD subjects compared to control subjects [20,35,36]. Some of these bacteria (such as *Prevotella* in rheumatoid arthritis and *Desulfovibrio* in IBD) have been associated with other non-infectious systemic inflammatory conditions [23,37]. 

## 3. Environmental Risk and the Microbiota in AMD: AREDS, Diet and Smoking

Dietary factors, oral supplements, and other environmental risk factors such as smoking may promote AMD through their effects on the intestinal microbiota. While analysis of the role of nutritional factors is notoriously difficult, an oral supplement containing the carotenoids lutein and zeaxanthin, or beta-carotene (original formulation), minerals, and antioxidants was found by the Age-Related Eye Disease Studies (AREDS and AREDS2) to reduce the risk of AMD progression (in up to 35% of patients) and remains the only known therapy for primary prevention of advanced disease [38]. How AREDS supplementation slows progression to advanced AMD is not completely understood. Beta-carotene is a chemical precursor to the in vivo production of vitamin A, a molecule that is central to the visual cycle that occurs in photoreceptor and RPE cells, whereas lutein and zeaxanthin form macular pigments that reduce photooxidative damage to the RPE. These carotenoids are not produced by most animals, including humans, and thus must be consumed through the diet; however, carotenoids are in fact produced by certain bacteria that contain carotenogenic genes. Carotenoids are also broken down by bacteria that harbor carotenoid cleavage enzymes [39,40]. Thus, it is plausible that the bioavailability of carotenoids is altered by the composition of the intestinal microbiota depending on their ability to produce or break down these biochemicals. Bacteria are also known to alter bile acid concentrations in the gut, which can affect carotenoid bioavailability [41]. Lin and colleagues have shown that AREDS supplementation has a significant impact on intestinal bacteria populations, causing enhancement of alpha diversity (a measure of bacterial species evenness and richness) [36]. Additionally, bacterial carotenoid biosynthesis pathways, as determined by inference from the intestinal bacterial sequencing data, were indeed influenced significantly in advanced AMD either due to AREDS, which was commonplace in AMD subjects, or due to the disease state [42].

Other dietary factors may also affect AMD pathogenesis and progression via interactions with the intestinal microbiota. Andriessen and colleagues investigated a murine model of choroidal neovascularization in which a high fat diet resulted in increased weight gain, alterations in the composition of the microbiome, increased intestinal permeability, and promotion of CNV growth compared to mice on a regular diet. Weight gain persisted after administration of oral antibiotics, but CNV growth slowed, suggesting that gut microbial alterations and not simply obesity were responsible for angiogenesis promotion [6]. Skondra and colleagues also found that a high fat diet induces an intestinal dysbiosis characterized by increased *Firmicutes* and *Verrucomicrobia* phyla and decreased *Bacteroides,* similar to Andreissen’s study. In this study, they describe high fat diet-induced dysbiosis concomitantly influences purine pathway in the gut- microbiota and in the retina in the same animals using multiomics studies [43]. Interestingly, changes in the microbiome purine signaling pathways were also associated with C3-deficiency in mice and neovascular AMD, highlighting a possible association between the intestinal microbiome and the complement system in neovascular AMD [44].Mice deficient in C3 also had a disturbed *Firmicutes* to *Bacteroides* ratio [44], similar to animals treated with the high-fat diet in both Andriessen’s [6] and Skondra’s studies [43]. Further, Zhang and colleagues have demonstrated that a high-fat diet in peroxisome proliferator-activated receptor-γ coactivator 1α (PGC-1α) +/- mice (which express lower levels of PGC-1α) causes the development of RPE and retinal morphological changes akin to an AMD-like phenotype, representing potentially microbiota-independent effects, although the latter study did not test the influence of the microbiota [45]. 

Rowan and colleagues demonstrated that mice given a high-sugar diet developed an altered intestinal microbiota along with AMD-like characteristics, such as loss of photoreceptors, retinal pigment epithelial atrophy, and sub-RPE deposits, as well as buildup of lipofuscin. On the other hand, a low sugar diet did not impact the retina nor the intestinal microbiota in this manner [46]. This same group additionally discovered that specific intestinal microbial products, such as serotonin (a neurotransmitter), shielded against an AMD-similar phenotype [46].

Interestingly, omega-3 fatty acids, at the doses used in the AREDS2 study, were not found to prevent progression to advanced AMD by oral supplementation. However, they were shown in epidemiologic studies to be beneficial in AMD, particularly when consumed as omega-3 fatty acid-rich fish [4,38]. One potential mechanism for omega-3 fatty acid protection in AMD is its action on the microbiota. After oral omega-3 fatty acid supplementation, it has been demonstrated that there can be an increase in beneficial intestinal bacteria, such as butyrate-producing *Lachnospiraceae* [47]. Butyrate is one of the colonic short chain fatty acids thought to be important in regulating the immune system as well as maintaining intestinal epithelial barrier integrity, thus reducing inflammation in the gut and elsewhere in the body, including ocular inflammation [28,48]. 

Finally, the composition of the microbiota can be heavily influenced by exposure to oxidants from smoking and other environmental sources. Such exposure may result in a pro-inflammatory repertoire of commensal bacteria [49], a dysbiosis that could possibly be reversed by antioxidants taken through the diet or via oral supplementation.

## 4. Genes and Gene Expression in AMD: A Link to the Microbiota 

The most robust genome-wide association study (GWAS) in AMD completed up to the present time exhibited 52 genetic variants (34 loci) significantly associated with AMD [8]. Gene variants in the complement factor H (*CFH*) and *CFI* genes, both inhibitors of complement activation, were strongly associated with AMD. A gene variant in the *ARMS2* gene, which may also be involved in the complement cascade, but is not well understood, is also highly associated with AMD. *LIPC*, APOE (lipid pathway genes), metalloproteinase (*TIMP3*), and other inflammatory pathway gene variants were associated with AMD. Both CFH and ARMS2 proteins are thought to be important in the alternative pathway of the complement cascade, and their deficiency may be responsible for reduced clearance of cellular debris and its accumulation beneath the RPE and along Bruch’s membrane in the form of drusen deposits [50]. These 52 genetic variants explain only approximately 50% of the genomic heritability of AMD. Thus, there are either currently unknown genetic variants or environmental causes that bring about dysfunctional complement activity and lipid/inflammatory pathway disruption. Studies have shown that many AMD patients exhibit increased serum complement activity even when lacking known complement pathway gene variants [10,51]. Furthermore, independently of lipid metabolism or complement pathway gene variants, the hallmark of AMD is the presence of complement, oxidized lipid, and other inflammatory-factor-laden deposits called drusen underneath the retina [10,51]. 

A dysbiosis of the gut or other part of the human body, resulting in inappropriate chronic over-activation of the complement cascade, could be one possible host environmental factor contributing to the pathogenesis of AMD. Lin and colleagues have found that genetic risk scores (calculated from 21 risk alleles associated with AMD) are inversely correlated with alpha diversity of the gut bacteria in advanced AMD patients, and there is a particularly strong correlation between *ARMS2* and *CFH* risk alleles and lowered alpha diversity. Additionally, we have found that the *ARMS2* risk allele was associated with significantly higher rates of IgA-bound bacteria in the gut, signifying the immunological relevance of these gut bacterial changes [36]. In a recent study by Zysset-Burri and colleagues, the bacteria *Negativicutes* was found to be a potential biomarker for nvAMD and also appeared to positively correlate with an AMD-associated *CFH* risk allele [44]. 

A synergistic effect of genetic predisposition, high-fat diet, and aging has been revealed by the work of Skondra and colleagues using AMD animal models. A high-fat diet accelerated and exacerbated both wet- and dry-AMD features in genetically predisposed apolipoprotein-E deficient mice more robustly compared to wild-type mice [52]. Microbiome–host interactions are very complex, and thus specialized animal models are needed in order to answer the fundamental questions on the relationship between the gut microbiome and disease states to delineate these interactions. Germ-free (GF) and gnotobiotic animal models, in which GF mice of different genetic backgrounds are colonized with specific microbial populations or strains, are considered the gold-standard animal models for microbiome studies. Development and utilization of these animal models has led to significant discoveries for the crucial role of the gut microbiome in inflammatory bowel diseases and in the gut–brain axis [53,54]. Studies on connections between the gut microbiome and retinal diseases have been focused mostly on animal models using antibiotics and diet to eliminate and alter the gut microbiome, respectively. The diet/antibiotic animal models are important and insightful for microbiome studies and are much more economically feasible, easier to use, and much more accessible than the GF models, which are considered the gold standard. Nonetheless, the antibiotic models have several disadvantages, including, but not limited to, off-target drug effects, alteration of host physiologic parameters, and a possible selection of resistant strains [55]. Our group has described the first GF mouse model of laser-induced choroidal neovascularization (CNV), a commonly used model of neovascular AMD [56]. Further studies using the above described GF-CNV model and expansion to more GF and gnotobiotic dry and neovascular AMD mouse models will be invaluable to delineate the precise microbiome–host interactions in AMD.

Using high-throughput RNA sequencing in GF mice, Skondra and colleagues has also revealed key aspects of retinal gene regulation important in AMD pathogenesis that are modulated by the intestinal microbiota. Their study revealed that the top modified pathways include vascular endothelial growth factor (VEGF) receptor signaling, mitochondrial biogenesis, oxidative stress, autophagy, longevity, and AMP-activated protein kinase (AMPK) signaling [57]. In the same study, one of the top differentially expressed genes of the gut–retina axis was revealed to be the peroxisome proliferator-activated receptor gamma coactivator 1-alpha (PGC-1α), which is the master regulator of mitochondrial biogenesis, oxidative stress, and lysosomal lipid trafficking [58,59,60]. Using RPE from human donor AMD eyes, dysregulation of AMPK/PGC-1α and mitochondrial biogenesis has been shown to play a key role in AMD pathogenesis [61]. The similarity between known dysregulated genes, biologic pathways in AMD pathogenesis, and those found to be important in the gut–retina axis implies an intimate connection between the two.

## 5. Inflammation and the Microglia in AMD: The Role of the Gut Microbiome 

Dysregulated immune activation via recruitment of microglia and macrophages into the subretinal and choroidal areas, mast cell activation, and RPE immune activation all likely play a role in AMD pathogenesis or progression [62]; however, the origin of this inflammation remains nebulous. Emerging evidence has delineated mechanisms of intestinal microbiota-induced-microglia activation, but the mechanism by which the microbiome modulates retinal microglial functions is not well understood [63,64]. Mononuclear phagocytes (microglia and macrophages) localize to the site of neovascularization in laser-induced CNV [65]. Andriessen and colleagues demonstrated that mice with an intestinal dysbiosis secondary to a high-fat diet have a two-fold increase in the localization of both microglia and macrophages within CNV lesions, compared to normal-diet-fed mice, and that this increase was mitigated by eradicating the gut-microbiota via neomycin [6]. These observations support a contributory role of the intestinal microbiota in neovascular AMD pathogenesis by promoting low grade inflammation. More importantly, these findings suggest that these deleterious effects can be modified by therapeutically targeting the microbiota. In fact, supplementation with the probiotic strain, *L.paracasei KW3110,* a lactic acid-producing bacteria that can activate macrophages but paradoxically suppresses inflammation, leads to an altered Firmicutes/Bacteroidetes ratio [66]. Several studies have shown that there is an abnormal Firmicutes/Bacteroidetes ratio in AMD [6,44]. Feeding aged mice heat-killed *L.paracasei* resulted in a reversal of the deleterious Firmicutes/Bacteroidetes ratio and decreased both age-related inflammation and ganglion cell loss in the retina [66]. 

## 6. Conclusions

**Figure 1:**
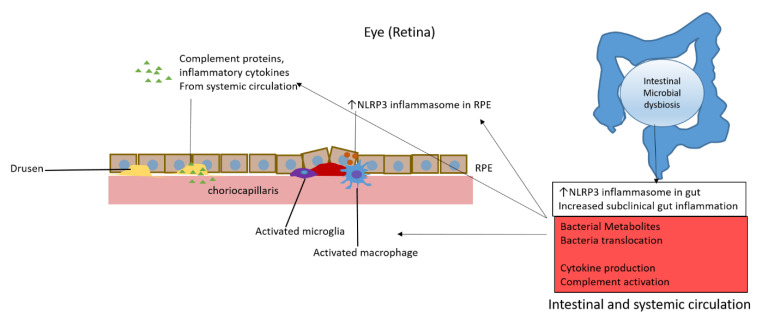
Potential mechanisms of intestinal dysbiosis promotion of age-related macular degeneration through activation of complement, microglia and macrophages, inflammatory cytokines, and inflammasomes.

Several groups have found that an intestinal dysbiosis occurs in AMD patients, and that certain dietary changes can cause AMD-like phenotypes due to changes in the microbiota. Centralized repositories analyzing bacterial sequencing and patient metadata, as well as a multi-centered effort (similar to the GWAS effort), are required to establish a clearer pattern of distinct microbial profiles that might be used as biomarkers for disease development and severity. Additional work proving the implication that AREDS and dietary changes may be beneficial in preventing the progression of AMD through their action on the intestinal microbiota, and will require experimentation in both animal models and longitudinal data collection in patients. Future studies using specialized GF and gnotobiotic animal models will be essential to delineate the interaction of the gut microbiome with other complex host factors in AMD pathogenesis as well as progression. The association between microbial changes and the genetic risk alleles of the complement pathway genes associated with AMD provide clues to what may be promoting non-genetic risk allele AMD patients to have aberrantly elevated systemic complement activation. As pointed out by Handa et al. [62], the links between genetic, non-genetic and environmental/lifestyle factors of AMD have been examined in a reductionist manner which might be insufficient to find the effect between unrelated, but possibly synergistic factors. Though the relative effects of the gut microbiome, genetics, and diet on AMD are now being studied in terms of changes on the host immune system, a systems biology approach integrating genomic, proteomic, metabolomic, medical, pharmacological, and clinical data is needed to achieve a deeper understanding of these complex interactions. We now know that a multitude of inflammatory mechanisms known to be influential in AMD pathogenesis can be influenced by changes in the intestinal microbiota (see Figure 1). Thus, intestinal microbiota and their products of metabolism likely have a large impact on both AMD pathogenesis and progression, and they may offer a potential direct site for novel therapeutic targeting. 

## Data Availability

Not applicable.

## References

[1] Pennington K.L., DeAngelis M.M. (2016). Epidemiology of age-related macular degeneration (AMD): Associations with cardiovascular disease phenotypes and lipid factors. Eye Vis..

[2] Rein D.B., Wittenborn J.S., Zhang X., Honeycutt A.A., Lesesne S.B., Saaddine J. (2009). Vision Health Cost-Effectiveness Study Group. Forecasting age-related macular degeneration through the year 2050: The potential impact of new treatments. Arch. Ophthalmol..

[3] Chakravarthy U., Wong T.Y., Fletcher A., Piault E., Evans C., Zlateva G., Buggage R., Pleil A., Mitchell P. (2010). Clinical risk factors for age-related macular degeneration: A systematic review and meta-analysis. BMC Ophthalmol..

[4] Chapman N.A., Jacobs R.J., Braakhuis A.J. (2019). Role of diet and food intake in age-related macular degeneration: A systematic review. Clin. Exp. Ophthalmol..

[5] Adams M.K.M., Simpson J.A., Aung K.Z., Makeyeva G.A., Giles G.G., English D.R., Hopper J., Guymer R.H., Baird P.N., Robman L.D. (2011). Abdominal Obesity and Age-related Macular Degeneration. Am. J. Epidemiol..

[6] Andriessen E.M., Wilson A.M., Mawambo G., Dejda A., Miloudi K., Sennlaub F., Sapieha P. (2016). Gut microbiota influences pathological angiogenesis in obesity-driven choroidal neovascularization. EMBO Mol. Med..

[7] Gottfredsdottir M.S., Sverrisson T., Musch D.C., Stefánsson E. (1999). Age related macular degeneration in monozygotic twins and their spouses in Iceland. Acta Ophthalmol. Scand..

[8] Fritsche L.G., Igl W., Bailey J.N.C., Grassmann F., Sengupta S., Bragg-Gresham J.L., Burdon K.P., Hebbring S.J., Wen C., Gorski M. (2016). A large genome-wide association study of age-related macular degeneration highlights contributions of rare and common variants. Nat. Genet..

[9] Crabb J.W., Miyagi M., Gu X., Shadrach K., West K.A., Sakaguchi H., Kamei M., Hasan A., Yan L., Rayborn M.E. (2002). Drusen proteome analysis: An approach to the etiology of age-related macular degeneration. Proc. Natl. Acad. Sci. USA.

[10] Scholl H.P.N., Issa P.C., Walier M., Janzer S., Pollok-Kopp B., Börncke F., Fritsche L.G., Chong N.V., Fimmers R., Wienker T. (2008). Systemic complement activation in age-related macular degeneration. PLoS ONE.

[11] Doyle S.L., Campbell M., Ozaki E., Salomon R.G., Mori A., Kenna P.F., Farrar G.J., Kiang A.-S., Humphries M.M., Lavelle E.C. (2012). NLRP3 has a protective role in age-related macular degeneration through the induction of IL-18 by drusen components. Nat. Med..

[12] Tarallo V., Hirano Y., Gelfand B.D., Dridi S., Kerur N., Kim Y., Gil Cho W., Kaneko H., Fowler B.J., Bogdanovich S. (2012). DICER1 Loss and Alu RNA Induce Age-Related Macular Degeneration via the NLRP3 Inflammasome and MyD88. Cell.

[13] Ardeljan D., Wang Y., Park S., Shen D., Chu X.K., Yu C.-R., Abu-Asab M., Tuo J., Eberhart C.G., Olsen T.W. (2014). Interleukin-17 Retinotoxicity Is Prevented by Gene Transfer of a Soluble Interleukin-17 Receptor Acting as a Cytokine Blocker: Implications for Age-Related Macular Degeneration. PLoS ONE.

[14] Doyle S.L., Ozaki E., Brennan K., Humphries M.M., Mulfaul K., Keaney J., Kenna P.F., Maminishkis A., Kiang A.-S., Saunders S.P. (2014). IL-18 Attenuates Experimental Choroidal Neovascularization as a Potential Therapy for Wet Age-Related Macular Degeneration. Sci. Transl. Med..

[15] Seddon J.M., George S., Rosner B., Rifai N. (2005). Progression of age-related macular degeneration: Prospective assessment of C-reactive protein, interleukin 6, and other cardiovascular biomarkers. Arch. Ophthalmol..

[16] Cao X., Shen D., Patel M.M., Tuo J., Johnson T.M., Olsen T.W., Chan C.-C. (2011). Macrophage polarization in the maculae of age-related macular degeneration: A pilot study. Pathol. Int..

[17] Combadière C., Feumi C., Raoul W., Keller N., Rodéro M., Pézard A., Lavalette S., Houssier M., Jonet L., Picard E. (2007). CX3CR1-dependent subretinal microglia cell accumulation is associated with cardinal features of age-related macular degeneration. J. Clin. Investig..

[18] Gupta N., Brown K.E., Milam A.H. (2003). Activated microglia in human retinitis pigmentosa, late-onset retinal degeneration, and age-related macular degeneration. Exp. Eye Res..

[19] Costello M.-E., Ciccia F., Willner D., Warrington N.M., Robinson P.C., Gardiner B., Marshall M., Kenna T.J., Triolo G., Brown M.A. (2015). Brief Report: Intestinal Dysbiosis in Ankylosing Spondylitis. Arthritis Rheumatol..

[20] Lin P. (2019). Importance of the intestinal microbiota in ocular inflammatory diseases: A review. Clin. Exp. Ophthalmol..

[21] Manasson J., Shen N., Ferrer H.R.G., Ubeda C., Iraheta I., Heguy A., Von Feldt J.M., Espinoza L.R., Kutzbach A.G., Segal L.N. (2018). Gut Microbiota Perturbations in Reactive Arthritis and Postinfectious Spondyloarthritis. Arthritis Rheumatol..

[22] Miyake S., Kim S., Suda W., Oshima K., Nakamura M., Matsuoka T., Chihara N., Tomita A., Sato W., Kim S.-W. (2015). Dysbiosis in the Gut Microbiota of Patients with Multiple Sclerosis, with a Striking Depletion of Species Belonging to Clostridia XIVa and IV Clusters. PLoS ONE.

[23] Scher J.U., Sczesnak A., Longman R.S., Segata N., Ubeda C., Bielski C., Rostron T., Cerundolo V., Pamer E.G., Abramson S.B. (2013). Expansion of intestinal Prevotella copri correlates with enhanced susceptibility to arthritis. eLife.

[24] Vaarala O. (2012). Gut Microbiota and Type 1 Diabetes. Rev. Diabet. Stud..

[25] Wang Z., Klipfell E., Bennett B.J., Koeth R., Levison B.S., Dugar B., Feldstein A.E., Britt E.B., Fu X., Chung Y.-M. (2011). Gut flora metabolism of phosphatidylcholine promotes cardiovascular disease. Nat. Cell Biol..

[26] Huang X., Ye Z., Cao Q., Su G., Wang Q., Deng J., Zhou C., Kijlstra A., Yang P. (2018). Gut Microbiota Composition and Fecal Metabolic Phenotype in Patients with Acute Anterior Uveitis. Investig. Opthalmol. Vis. Sci..

[27] Janowitz C., Nakamura Y.K., Metea C., Gligor A., Yu W., Karstens L., Rosenbaum J.T., Asquith M., Lin P. (2019). Disruption of Intestinal Homeostasis and Intestinal Microbiota During Experimental Autoimmune Uveitis. Investig. Opthalmol. Vis. Sci..

[28] Nakamura Y.K., Janowitz C., Metea C., Asquith M., Karstens L., Rosenbaum J.T., Lin P. (2017). Short chain fatty acids ameliorate immune-mediated uveitis partially by altering migration of lymphocytes from the intestine. Sci. Rep..

[29] Nakamura Y.K., Metea C., Karstens L., Asquith M., Gruner H., Moscibrocki C., Lee I., Brislawn C.J., Jansson J.K., Rosenbaum J.T. (2016). Gut Microbial Alterations Associated With Protection From Autoimmune Uveitis. Investig. Opthalmol. Vis. Sci..

[30] Horai R., Zárate-Bladés C.R., Dillenburg-Pilla P., Chen J., Kielczewski J.L., Silver P.B., Jittayasothorn Y., Chan C.-C., Yamane H., Honda K. (2015). Microbiota-Dependent Activation of an Autoreactive T Cell Receptor Provokes Autoimmunity in an Immunologically Privileged Site. Immunity.

[31] Chen H., Cho K.S., Vu T.K., Shen C.H., Kaur M., Chen G., Mathew R., McHam M.L., Fazelat A., Lashkari K. (2018). Commensal microflora-induced T cell responses mediate progressive neurodegeneration in glaucoma. Nat. Commun..

[32] Beli E., Yan Y., Moldovan L., Vieira C.P., Gao R., Duan Y., Prasad R., Bhatwadekar A., White F.A., Townsend S.D. (2018). Restructuring of the Gut Microbiome by Intermittent Fasting Prevents Retinopathy and Prolongs Survival indb/dbMice. Diabetes.

[33] Skondra D., Rodriguez S.H., Sharma A., Gilbert J., Andrews B., Claud E.C. (2020). The early gut microbiome could protect against severe retinopathy of prematurity. J. Am. Assoc. Pediatr. Ophthalmol. Strabismus.

[34] Zinkernagel M.S., Zysset-Burri D.C., Keller I., Berger L.E., Leichtle A.B., Largiadèr C.R., Fiedler G.M., Wolf S. (2017). Association of the Intestinal Microbiome with the Development of Neovascular Age-Related Macular Degeneration. Sci. Rep..

[35] Lin P. (2018). The role of the intestinal microbiome in ocular inflammatory disease. Curr. Opin. Ophthalmol..

[36] Lin P., McClintic S.M., Saleh M.G., Kiang L., Karsten L.J., Asquith M., Martin T.M., Klein M.J. AREDS supplementation and genetic risk score are crucial determinants of microbial alterations in advanced age-related macular degeneration, in preparation.

[37] Berry D., Reinisch W. (2013). Intestinal microbiota: A source of novel biomarkers in inflammatory bowel diseases?. Best Pr. Res. Clin. Gastroenterol..

[38] Chew E.Y., Clemons T.E., Agrón E., Launer L.J., Grodstein F., Bernstein P.S. (2015). Effect of omega-3 fatty acids, lutein/zeaxanthin, or other nutrient supplementation on cognitive function: The AREDS2 randomized clinical trial. JAMA.

[39] Ahrazem O., Gómez-Gómez L., Rodrigo M.J., Avalos J., Limón M.C. (2016). Carotenoid Cleavage Oxygenases from Microbes and Photosynthetic Organisms: Features and Functions. Int. J. Mol. Sci..

[40] Liang M.-H., Zhu J., Jiang J.-G. (2017). Carotenoids biosynthesis and cleavage related genes from bacteria to plants. Crit. Rev. Food Sci. Nutr..

[41] Bohn T., Desmarchelier C., Dragsted L.O., Nielsen C.S., Stahl W., Rühl R., Keijer J., Borel P. (2017). Host-related factors explaining interindividual variability of carotenoid bioavailability and tissue concentrations in humans. Mol. Nutr. Food Res..

[42] Kiang L., McClintic S., Saleh M., Metea C., Mitio K., Asquith M., Martin T.M., Klein M.L., Karstens L., Lin P. (2017). The gut microbiome in advanced age-related macular degeneration. Investig. Ophthalmol. Vis. Sci..

[43] Skondra D., Xie B., Nadeem U., Movahedan A., D’Souza M., Barba H., Deng N., Leone V.A., Chang E., Sulakhe D. Using integrated multiomics approach to describe the role of high-fat diet induced gut-dysbiosis in age-related macular degeneration, in preparation.

[44] Zysset-Burri D.C., Keller I., Berger L.E., Largiadèr C.R., Wittwer M., Wolf S., Zinkernagel M.S. (2020). Associations of the intestinal microbiome with the complement system in neovascular age-related macular degeneration. NPJ Genom. Med..

[45] Zhang M., Chu Y., Mowery J., Konkel B., Galli S., Theos A.C., Golestaneh N. (2018). Pgc-1α repression and high-fat diet induce age-related macular degeneration-like phenotypes in mice. Dis. Model. Mech..

[46] Rowan S., Jiang S., Korem T., Szymanski J., Chang M.-L., Szelog J., Cassalman C., Dasuri K., McGuire C., Nagai R. (2017). Involvement of a gut–retina axis in protection against dietary glycemia-induced age-related macular degeneration. Proc. Natl. Acad. Sci. USA.

[47] Costantini L., Molinari R., Farinon B., Merendino N. (2017). Impact of Omega-3 Fatty Acids on the Gut Microbiota. Int. J. Mol. Sci..

[48] Smith P.M., Howitt M.R., Panikov N., Michaud M., Gallini C.A., Bohlooly-Y M., Glickman J.N., Garrett W.S. (2013). The Microbial Metabolites, Short-Chain Fatty Acids, Regulate Colonic Treg Cell Homeostasis. Science.

[49] Savin Z., Kivity S., Yonath H., Yehuda S. (2018). Smoking and the intestinal microbiome. Arch. Microbiol..

[50] Micklisch S., Lin Y., Jacob S., Karlstetter M., Dannhausen K., Dasari P., Von Der Heide M., Dahse H.-M., Schmölz L., Grassmann F. (2017). Age-related macular degeneration associated polymorphism rs10490924 in ARMS2 results in deficiency of a complement activator. J. Neuroinflamma..

[51] Hecker L.A., Edwards A.O., Ryu E., Tosakulwong N., Baratz K.H., Brown W.L., Issa P.C., Scholl H.P., Pollok-Kopp B., Schmid-Kubista K.E. (2009). Genetic control of the alternative pathway of complement in humans and age-related macular degeneration. Hum. Mol. Genet..

[52] Skondra D., She H., Zambarakji H.J., Connolly E., Michaud N., Chan P., Kim I.K., Gragoudas E.S., Miller J.W., Hafezi-Moghadam A. (2007). Effects of ApoE Deficiency, Aging and High Fat Diet on Laser-Induced Choroidal Neovascularization and Bruch’s Membrane-RPE Interface Morphology. Investig. Ophthalmol. Vis. Sci..

[53] Miyoshi J., Chang E.B. (2017). The gut microbiota and inflammatory bowel diseases. Transl. Res..

[54] Sudo N., Chida Y., Aiba Y., Sonoda J., Oyama N., Yu X.-N., Kubo C., Koga Y. (2004). Postnatal microbial colonization programs the hypothalamic-pituitary-adrenal system for stress response in mice. J. Physiol..

[55] Ericsson A.C., Franklin C.L. (2015). Manipulating the Gut Microbiota: Methods and Challenges. ILAR J..

[56] Dimitra Skondra M.D., Movahedan A., Spedale M., Deng N., Leone V., Chang E., Theriault B. (2019). Gnotobiotic Animal Model of Laser-Induced Choroidal Neovascularization in Germ-Free Mice. Investig. Ophthalmol Vis. Sci..

[57] Nadeem U., Xie B., Movahedan A., D’Souza M., Barba H., Deng N., Leone V.A., Chang E., Sulakhe D., Skondra D. (2020). High Throughput RNA Sequencing of Mice Retina Reveals Metabolic Pathways Involved in the Gut-Retina Axis. bioRxiv.

[58] Handschin C., Spiegelman B.M. (2006). Peroxisome proliferator-activated receptor gamma coactivator 1 coactivators, energy homeostasis, and metabolism. Endocr. Rev..

[59] Lin J., Handschin C., Spiegelman B.M. (2005). Metabolic control through the PGC-1 family of transcription coactivators. Cell Metab..

[60] Vainshtein A., Tryon L.D., Pauly M., Hood D.A. (2015). Role of PGC-1α during acute exercise-induced autophagy and mitophagy in skeletal muscle. Am. J. Physiol. Physiol..

[61] Zhang M., Jiang N., Chu Y., Postnikova O., Varghese R., Horvath A., Cheema A.K., Golestaneh N. (2020). Dysregulated metabolic pathways in age-related macular degeneration. Sci. Rep..

[62] Handa J.T., Rickman C.B., Dick A.D., Gorin M.B., Miller J.W., Toth C.A., Ueffing M., Zarbin M., Farrer L.A. (2019). A systems biology approach towards understanding and treating non-neovascular age-related macular degeneration. Nat. Commun..

[63] Erny D., De Angelis A.L.H., Jaitin D., Wieghofer P., Staszewski O., David E., Keren-Shaul H., Mahlakoiv T., Jakobshagen K., Buch T. (2015). Host microbiota constantly control maturation and function of microglia in the CNS. Nat. Neurosci..

[64] Mosher K.I., Wyss-Coray T. (2015). Go with your gut: Microbiota meet microglia. Nat. Neurosci..

[65] Lückoff A., Scholz R., Sennlaub F., Xu H., Langmann T. (2017). Comprehensive analysis of mouse retinal mononuclear phagocytes. Nat. Protoc..

[66] Morita Y., Jounai K., Sakamoto A., Tomita Y., Sugihara Y., Suzuki H., Ohshio K., Otake M., Fujiwara D., Kanauchi O. (2018). Long-term intake of Lactobacillus paracasei KW3110 prevents age-related chronic inflammation and retinal cell loss in physiologically aged mice. Aging.

